# Binder Jetting Additive Manufacturing of Inconel 718/TiC Metal Matrix Composites: Influence of TiC Content on Processing, Microstructure, Mechanical and Tribological Properties

**DOI:** 10.3390/ma17205050

**Published:** 2024-10-16

**Authors:** Artem Borisov, Aleksey Shamshurin, Mark Kovalev, Anatoliy Popovich, Vadim Sufiiarov

**Affiliations:** 1 Institute of Machinery, Materials, and Transport, Peter the Great St. Petersburg Polytechnic University, 195251 St. Petersburg, Russia; 2 Institute of Advanced Manufacturing Technologies, Peter the Great St. Petersburg Polytechnic University, 195251 St. Petersburg, Russia

**Keywords:** additive manufacturing, binder jetting, metal matrix composite, inconel 718, titanium carbide, mechanical properties, wear resistance

## Abstract

This paper investigated the influence of titanium carbide (TiC) content on the processing, microstructure, mechanical and tribological properties of Inconel 718/TiC composites produced by binder jetting additive manufacturing. It was found that increasing the amount of TiC required an increase of the drying intensity during printing due to a decrease in the thermal conductivity of the powder mixture. The sintering process also depended on the TiC content. The most optimal modes were 1270 °C for 10 h for samples with 0 and 3% TiC and 1280 °C for 5 h for samples with 5 and 10% TiC. The hardness of the materials increased as the proportion of reinforcement increased. The best tensile properties, also at high temperatures, were possessed by samples with 3% TiC, showing high strength and, in addition, satisfactory plasticity. The maximum wear resistance was achieved by the composite material containing 5% TiC.

## 1. Introduction

Nickel alloys such as Inconel 718 are widely used in various engineering applications, especially in the aerospace industry, due to their combination of high strength, effective fatigue and creep resistance, as well as oxidation resistance at elevated temperatures and stable chemical properties at room temperature [1,2]. However, parts made of traditional nickel alloys often do not meet the increasing requirements, especially those related to wear resistance. In this regard, research aimed at solving this problem, including the development of metal matrix composites (MMC) with improved performance characteristics, is currently underway [3,4,5].

Metal matrix composites represent a distinctive category of materials that combine several heterogeneous materials, most often two—metal matrix and ceramic reinforcing components [6]. The metal matrix in such systems provides the necessary plasticity for the final material, while reinforcement with ceramics causes a significant increase in hardness and stiffness. Combining them creates materials with a complex of unique properties, namely, low specific weight, high strength characteristics, including at elevated temperatures, and resistance to abrasive wear [7,8,9,10].

The capacity of the reinforcing phase to enhance the characteristics of the metal matrix is contingent upon a number of factors, including its morphology, composition, distribution and volume fraction [11]. Reinforcing components can be used in the form of particles, short fibers or whiskers, as well as continuous fibers [12]. The use of continuous fibers in MMCs is significantly more expensive and also leads to anisotropic mechanical properties compared to the use of short fibers or particles [13], so reinforcement using ceramic particles is preferred for many applications [14]. Typically, ceramic reinforcing particles such as TiB_2_, TiC, TiB, Al_2_O_3_ and WC are introduced into an Inconel 718 matrix due to their high melting point, high elastic modulus, excellent wear resistance and good wettability between the particles and the metal matrix [15,16].

There are various types of technological strategies for the fabrication of MMCs, including both liquid-phase and solid-phase processes [17]. However, the use of liquid-phase methods often leads to inhomogeneous distribution of reinforcing phases [18], therefore, solid-phase processing methods are favored. The most common conventional methods of fabrication of MMCs are stir casting and powder metallurgy methods such as metal injection molding (MIM) [13,19,20]. At the same time, the use of casting for the production of MMCs based on the superalloy Inconel 718 can lead to the appearance of undesirable microstructural features, namely, the segregation of alloying elements occurring in the spaces between dendrites, contributing to the formation of harmful intermetallic compound–Laves phase, the elimination of which is difficult and requires multi-stage heat treatment [21]. In addition, liquid-phase production technologies can promote the floating-up or coagulation of carbide particles due to density differences and the possibility of a reaction between the strengthening phase and the metal melt cannot be ruled out. Moreover, both previously mentioned methods for MMC fabrication require additional processing using subtractive methods to obtain products of the desired geometry [22]. In turn, the addition of reinforcing particles into the matrix often leads to a worsening of machinability due to a decrease in plasticity and an increase in the wear resistance of the composite [12], which results in an increase in the cost of the manufacturing process [23]. The principal limitation of binder jetting technology is the difficulty in achieving high-density products after sintering. However, this can be effectively addressed through an additional treatment, namely hot isostatic pressing, which eliminates porosity.

The paper [24] describes the fabrication of metal matrix composites based on nickel alloy Inconel 625 using the hot isostatic pressing method. Fine powders of silicon carbide (SiC) and titanium diboride (TiB_2_) in different volume ratios from 5 to 25 vol.% were used as a strengthening phase. All the obtained composites showed homogeneous and dense microstructures without any significant defects. The hardness and wear resistance of the composites were in direct dependence on the volume fraction of reinforcement. The Inconel 625/10% SiC composite was characterized as the most optimal variant in terms of mechanical and tribological properties, as well as machinability, and was successfully applied to manufacturing demonstration samples of turbopump mechanical seals which showed high geometric accuracy of the original design.

The use of additive technologies in the fabrication of MMCs provides an opportunity to create lightweight and cost-effective structures of complex geometries and to avoid most of the limitations associated with traditional manufacturing methods [13,25,26]. Notwithstanding the considerable body of research elucidating the process parameters, microstructural evolution, and mechanical properties of metal matrix composites fabricated via selective laser melting (SLM) [27,28], electron beam melting (EBM) [29], and direct laser deposition (DLD) [30], a conspicuous dearth of understanding persists regarding the binder jetting (BJ) technique. It has garnered significant attention in recent years for its efficacy in producing ceramic and metal products with complex geometries and tailored properties [31,32,33]. The application of binder jetting additive manufacturing, in turn, is particularly promising due to its high productivity, relatively lower production costs (both in terms of equipment cost and energy consumption), and the absence of thermal stresses in the material structure because the process does not involve melting [34,35,36].

The paper [11] presented a procedure of application of the binder jetting technology followed by reactive sintering for the synthesis of metal matrix composites based on the nickel alloy Inconel 625. During the reaction process, between the chromium included in the alloying elements of the matrix and the carbon from the binder, a Cr_3_C_2_ carbide phase was formed. This strengthening phase was distributed along the grain boundaries and formed an interconnected network uniformly covering the entire matrix. Significant amounts of small niobium and molybdenum carbides were also observed within the grains, indicating intense carbon diffusion during the reactive sintering process. Wear tests showed that the specific wear rate of the Inconel 625 alloy composite was reduced by an average of 64% compared to the unreinforced counterpart. During the tests, both the carbide reinforcing phase and the fine carbides uniformly distributed in the nickel matrix showed a tendency to crack, with surface fatigue cracking identified as the main wear mechanism. This phenomenon is explained by the morphology of the continuously distributed hardening phase, which prevented the removal of carbides using a typical plowing mechanism.

In a prior study [37], the influence of TiC particle size on the microstructure and mechanical properties of the Inconel 718/TiC composite material produced through binder jetting additive manufacturing was examined. The results showed that the addition of 1 wt% of micron-sized TiC particles leads to a significant increase in the mechanical properties of the Inconel 718 superalloy, which was confirmed by room temperature and elevated temperature tests. The incorporation of nano-sized TiC particles, however, resulted in a reduction in mechanical properties, which did not attain the values observed in Inconel 718 samples without TiC addition. It is likely that this was due to the positioning of the nano-sized TiC particles on the surface of the Inconel 718 particles in the initial powders, which impeded the sintering process.

The potential applications of these composites are dependent on the properties obtained, which are in turn influenced by the quantity of carbide reinforcement. They can be utilized in high-temperature environments, such as turbine blades, turbocharger rotors, and reactor components [38]. Additionally, they can be well-suited for applications where abrasion resistance is of paramount importance, including drilling tools and pump components [39]. The objective of the present study is to examine the impact of TiC content in Inconel 718/TiC composite materials on processing, microstructure, strength characteristics, hardness and wear resistance.

## 2. Materials and Methods

**Figure 1:**
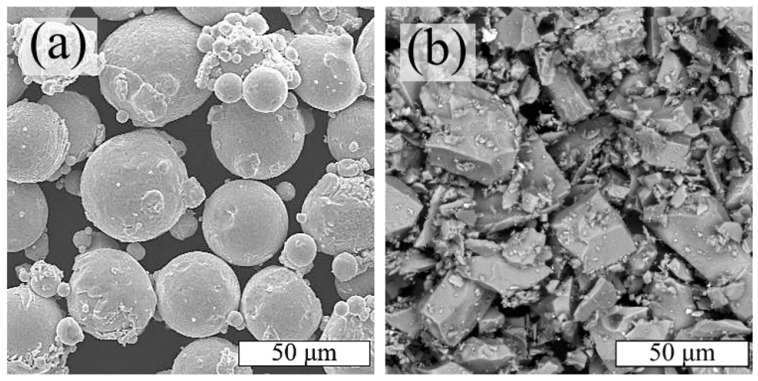
SEM images of powders: (**a**) Inconel 718; (**b**) TiC.

In the present study, the initial materials employed were Inconel 718 superalloy powder and titanium carbide (TiC) powder. Figure 1 illustrates the particle morphology of the initial powders.

The Inconel 718 powder was observed to exhibit a spherical shape, as well as the presence of satellites (Figure 1a), which is a characteristic feature of powders obtained through gas atomization [40]. The TiC powder exhibited a polyhedral particle morphology (Figure 1b).

Powder mixtures of Inconel 718 with the addition of varying quantities of TiC were prepared using a gravity mixer for a period of 12 h.

The particle size distribution of the powder was determined using laser diffraction using an Analysette 22 NanoTec plus analyzer (Fritsch GmbH, Idar-Oberstein, Germany), which is capable of measuring particle sizes in the range from 0.01 to 2000 µm.

The morphology of the powders and the microstructure of the obtained samples were studied using a scanning electron microscope (SEM) Tescan Mira3 LMU (TESCAN GROUP, Brno, Czech Republic) with backscattered electron (BSE) registration and an optical microscope Leica DMI5000 (Leica Microsystems GmbH, Wetzlar, Germany). A solution of nitric acid and hydrochloric acid in a 1:3 ratio was employed for the etching of the samples.

In order to analyze the grain size, the optical microscopy images were evaluated using the Image J software (ver. 1.54 g).

The samples for investigation were produced using the ExOne Innovent Binder Jetting System (The ExOne Company, North Huntington, PA, USA).

Phase analysis was conducted using a Bruker D8 Advance X-ray diffractometer (XRD) (Billerica, MA, USA) with Cu-Kɑ (1/4 1.5418 Å) irradiation.

Hot isostatic pressing (HIP) was conducted at 1160 ± 5 °C and 130 MPa pressure for a period of three hours. The samples were subjected to a heat treatment (HT) comprising annealing at 1060 ± 5 °C for one hour followed by air cooling and aging. The aging involved heating to 760 ± 5 °C and maintaining this temperature for ten hours, followed by cooling to 650 ± 5 °C for two hours and holding at 650 ± 5 °C for eight hours, after which the samples were cooled to room temperature under ambient conditions.

The densities of the sintered samples and samples subjected to HIP were determined using the Archimedes method in deionized water supplemented with a surfactant.

The hardness of the samples was determined utilizing a Zwick/Roell Zhu hardness tester (ZwickRoell GmbH & Co., Ulm, Germany) through the application of the Vickers method.

Tensile tests were conducted to ascertain the ultimate strength, yield strength and relative elongation of the samples. These tests were performed using Zwick/Roell z100 tensile testing machines at room temperature and a Zwick/Roell z050 machine at 700 ± 10 °C (ZwickRoell GmbH & Co., Ulm, Germany).

The tribological properties were evaluated in accordance with the ASTM G65 [41]. The test procedure entailed the abrasion of the tested surface of the samples with dry abrasive of a specified size and composition. Two test cycles were conducted in accordance with procedure B of the aforementioned standard (pressure load of 130 N and number of wheel revolutions of 2000). At the conclusion of each cycle, the mass loss of the specimens was quantified. The weighing was conducted on analytical scales with an accuracy class of 0.0001 g.

## 3. Results and Discussion

### 3.1. Initial Powders

The initial powder of Inconel 718 and its mixtures with TiC in the amounts of 3, 5 and 10 wt.% were investigated. The designations for the examined powders are outlined in Table 1.

**Figure 2:**
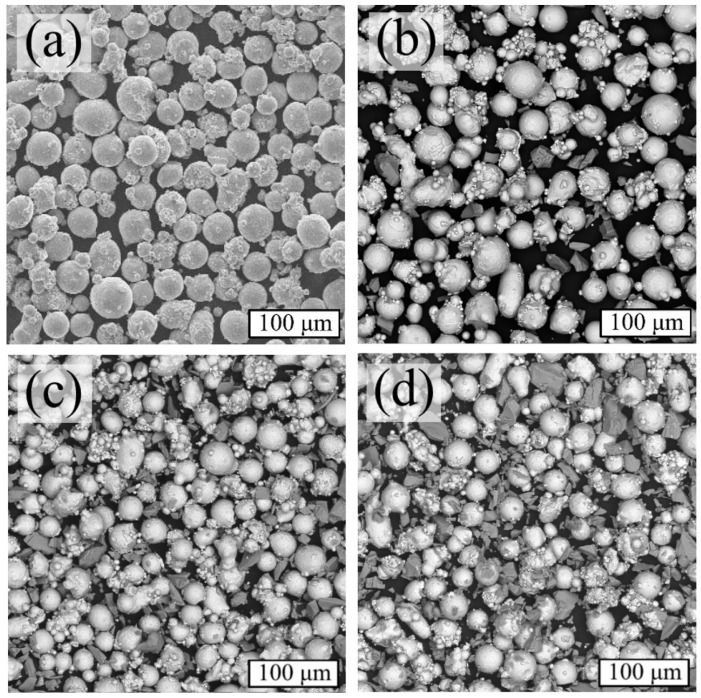
SEM images of powders (BSE): (**a**) Inc-0; (**b**) Inc-3; (**c**) Inc 5; (**d**) Inc-10.

The particle shape of the Inconel 718 powder could be characterized as rounded or spherical (Figure 2a), and the distribution of TiC particles in all powder mixtures as uniform, with a marked increase in its content (Figure 2b–d).

The particle size distribution of the powders is summarized in Table 2.

The average particle size decreased when TiC was added and when its content in the powder mixture was further increased. This was due to the fact that the TiC powder had a significantly smaller particle size compared to the Inconel 718 powder.

The spherical shape of the matrix powder particles is typically beneficial for the printing process as it facilitates the uniform application of powder layers due to the particles’ favorable flowability, which is a consequence of their morphology. Furthermore, the incorporation of smaller TiC particles can positively influence the density of the powder bed by filling the voids between the larger metal powder particles, which ultimately enhances the quality of the final product.

### 3.2. 3D Printing of Green Models

Binder jetting technology was used to obtain samples. Four groups of different types of blanks were produced: cubes—for density measurements, microstructure and phase composition studies, and hardness measurements; hexagonal prisms—for making samples and tensile tests; and cylinders—for abrasive wear tests. The printing parameters of samples made of various powder mixtures differed only in the conditions of the interlayer drying process: its temperature and time (Table 3).

The need to increase the drying intensity with increasing TiC content in the powder mixture was apparently due to the change in the physical properties of the powder. It is likely that as the TiC content increased, the thermal conductivity of the powder mixture decreased because TiC has a lower thermal conductivity than the Inconel 718 alloy, resulting in slower heat transfer and evaporation of the binder. To compensate for this phenomenon, it was necessary to use higher temperatures and longer drying times to sufficiently dry the binder. Based on preliminary test results, the printing parameters shown in Table 3 were the most suitable for each powder mixture in terms of obtaining the accurate geometry without significant printing defects and satisfactory density for the green model.

### 3.3. Sintering

Preliminary test sintering processes were conducted to determine the optimal sintering mode for samples of each of the four compositions. Mode variations included two sintering temperatures: 1270 °C and 1280 °C; and three different holding times: 3, 5, and 10 h. All sintering processes were carried out in a vacuum with a pre-stage of debinding at 600 °C for 1 h with argon blow.

**Figure 3:**
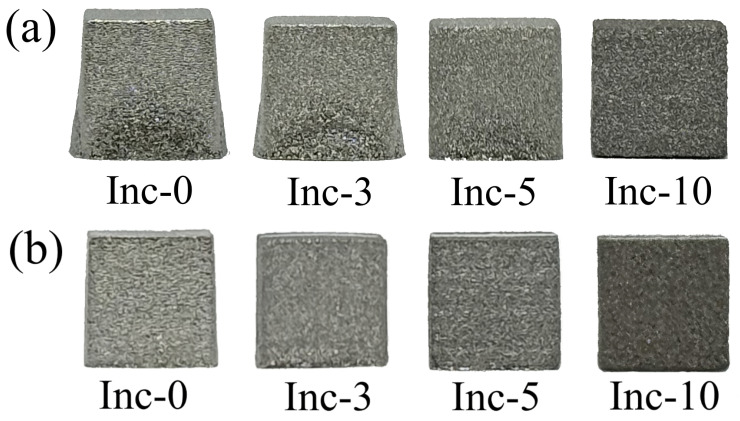
Images of samples sintered at: (**a**) 1280 °C for 3 h; (**b**) 1270 °C for 10 h.

As a result of sintering processes under test modes, it was found that when the sintering temperature was increased to 1280 °C, even at a minimum holding time of 3 h, Inc-0 and Inc-3 samples were characterized by distortion relative to the specified shape, namely, widening of the lower part of the samples as a result of liquid phase formation (Figure 3a), while all samples sintered at 1270 °C with a maximum holding time of 10 h fully retained the specified shape (Figure 3b).

The sintering process typically comprises several stages, with the driving force behind the process being the reduction of energy within the system, primarily through surface reduction. In the initial stages of sintering, interparticle contacts are formed and expand, while the porosity remains interconnected and open. Subsequently, a rapid reduction in volume occurs, resulting in the closure of the pores and their isolation from one another. In the final stage, the residual porosity gradually diminishes, accompanied by the coarsening of the grain. This is due to the fact that the pores that impede grain growth, despite their limited number, tend to vanish [42]. The application of excessive heat can result in the formation of a liquid phase, which may lead to distortion of the original shape of the product.

**Figure 4:**
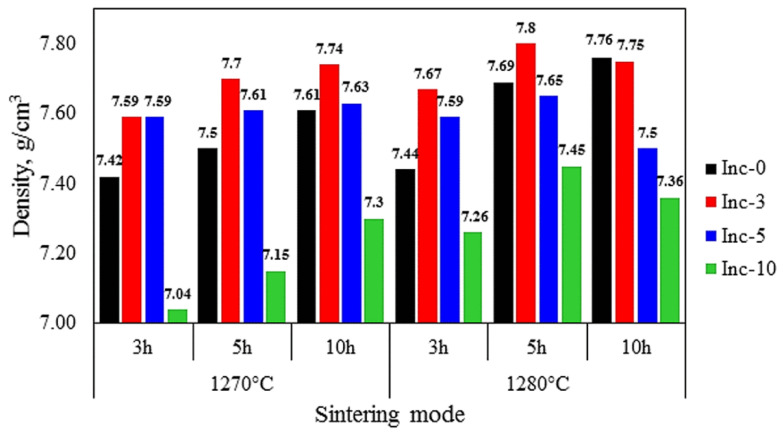
Density of sintered samples depending on the sintering mode.

The density was measured for all sintered samples; the results are shown in Figure 4.

Sintering at 1270 °C showed an increase in the density of Inc-0 and Inc-3 samples with increasing holding time, and the most successful mode in terms of density and preservation of the original geometry of these samples was sintering at 1270 °C for 10 h.

Inc-5 and Inc-10 samples had comparatively higher density values after sintering at 1280 °C and reached the highest values when held for 5 h. Subsequent increase in holding time up to 10 h resulted in a decrease in density in both types of samples. This was probably due to the so-called “over sintering” phenomenon, which describes the decrease in density and mechanical properties of a material during excessive sintering (excessive increase in temperature or holding time at sintering) because of intensive grain growth and pore coalescence [43].

**Figure 5:**
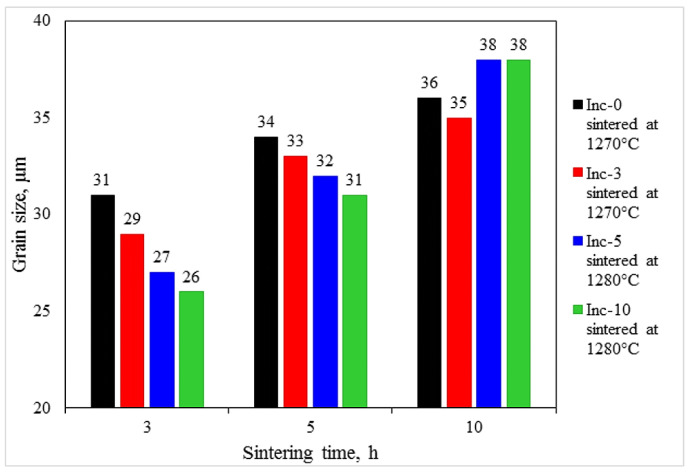
Grain size after sintering with constant temperature and different holding times: 3, 5 and 10 h.

The grain size was measured for samples sintered at a constant temperature (1270 °C for Inc-0 and Inc-3 samples and 1280 °C for Inc-5 and Inc-10 samples) but with different holding times. The results are presented in Figure 5.

**Figure 6:**
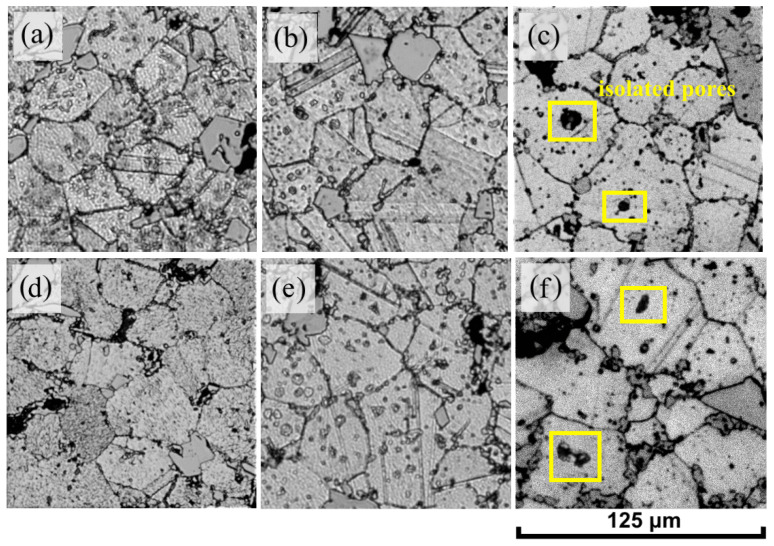
Microstructure of samples sintered at a constant temperature of 1280 °C for different times—3, 5 and 10 h, respectively: (**a**–**c**) Inc-5; (**d**–**f**) Inc-10.

For each single material, the average grain size increased with increasing holding time during sintering, with the largest grain enlargement observed when the holding time of Inc-5 and Inc-10 samples was increased to 10 h (Figure 6).

During the sintering process, pores and grain boundaries interact in three distinct ways. Firstly, pores can impede grain expansion. Secondly, they can become swept up by migrating grain boundaries as the grains grow. Thirdly, they can be left behind as grain boundaries detach and isolate them within the grain interior [44]. As the sintering temperature or holding time increases, the movement of grain boundaries may become more active [45]. Separation of the pores from the grain boundaries happens when the pores move at a slower pace than the grain boundaries, resulting in the formation of individual, isolated pores (Figure 6c,f). As the grains grow, the diffusion distance increases, making the elimination of isolated pores much more difficult because vacancies must diffuse to distant grain boundaries, which is a very slow process. In addition, the number of pores located at grain boundaries decreases and their size becomes larger [44]. The reason for this is that disparities in pore curvature drive a process where larger pores enlarge, while smaller, more unstable pores diminish, as governed by the Ostwald ripening mechanism [45]. Together, these phenomena lead to an increase in the overall porosity of the material, which explains the decrease in density at excessively long holding times during sintering.

The phenomenon of grain size increase in the Inc-5 and Inc-10 samples can be explained by the equilibrium between the processes of grain growth and the effects of particle consolidation under conditions of prolonged sintering. As exposure time increases, larger grains in the Inc-5 and Inc-10 samples are able to accumulate sufficient energy, enabling them to overcome or “drag” TiC particles along grain boundaries, thereby contributing to more intensive grain growth following attenuation of the anchoring effects.

**Figure 7:**
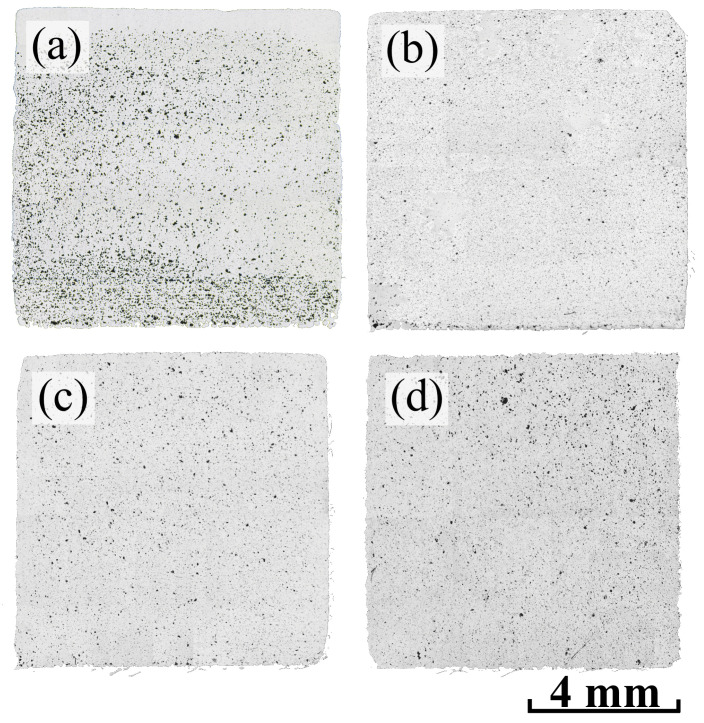
Panoramas of cross-sections of sintered samples: (**a**) Inc-0; (**b**) Inc-3; (**c**) Inc-5; (**d**) Inc-10.

Thus, the following modes were chosen for sintering the samples: a temperature of 1270 °C with 10 h of holding time for Inc-0 and Inc-3 samples and 1280 °C with 5 h of holding time for Inc-5 and Inc-10 samples. Panoramas of the samples sintered under these modes that show the porosity distribution along the cross-sections are presented in Figure 7.

### 3.4. Postprocessing, Microstructure Investigation and Tests

Despite the fact that the selected sintering modes were the most successful in terms of density and preservation of the sample geometry among all the modes tested in the preliminary tests, the samples were nevertheless characterized by a rather high pore content, and, in order to achieve decent mechanical properties, it was necessary to carry out post-processing, providing additional densification using hot isostatic pressing (HIP).

To evaluate the influence of HIP on the mechanical and tribological properties of each material, all samples were divided into 2 groups: in the first group, the sintered samples were subjected only to heat treatment (HT); in the second group, post-treatment of the samples included HIP and then HT. The modes of HT were identical for both groups of samples.

**Figure 8:**
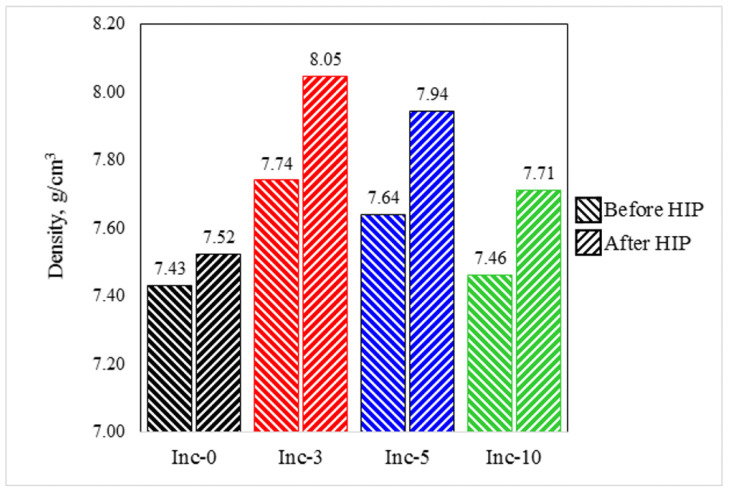
Comparison of the density of sintered samples before and after HIP.

The density of the samples was measured again after HIP. The results of the comparison of the density values before and after HIP for each material are presented in Figure 8.

All samples were characterized by some densification after HIP, but for sample Inc-0, the density increase after HIP was the least significant.

**Figure 9:**
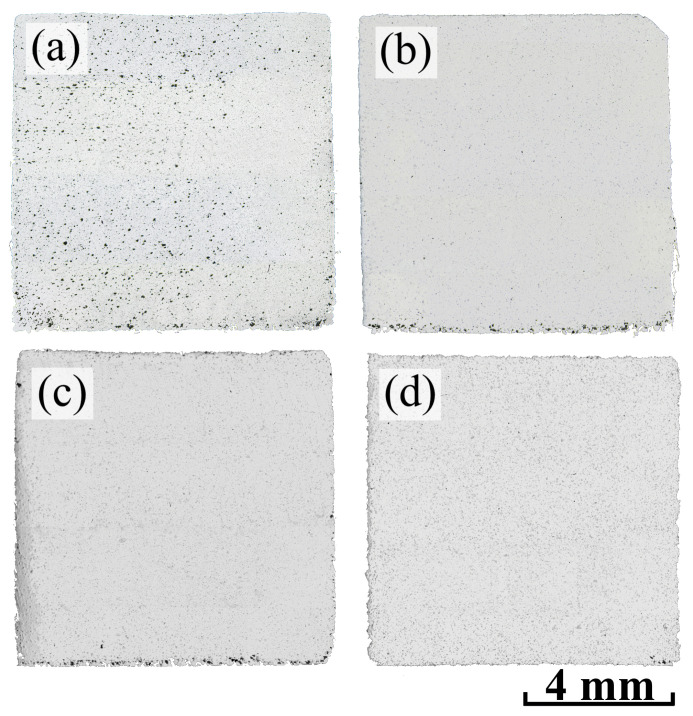
Panoramas of cross-sections of samples after HIP: (**a**) Inc-0; (**b**) Inc-3; (**c**) Inc-5; (**d**) Inc-10.

Panoramas of all samples after HIP are presented in Figure 9.

The cross-sections of samples Inc-3, Inc-5 and Inc-10 were characterized by the almost complete absence of pores, which is not the case for sample Inc-0, which has a large number of pores even after HIP. It is likely that the Inc-0 sample had open porosity, which the HIP had only a limited effect in reducing. Although the high pressure and temperature during the HIP process may contribute to some reduction of open porosity, the interconnected character of these pores makes it difficult to eliminate them completely. Open pores may still remain after HIP, especially if the material initially had a fairly high porosity and these pores were large [46,47].

**Figure 10:**
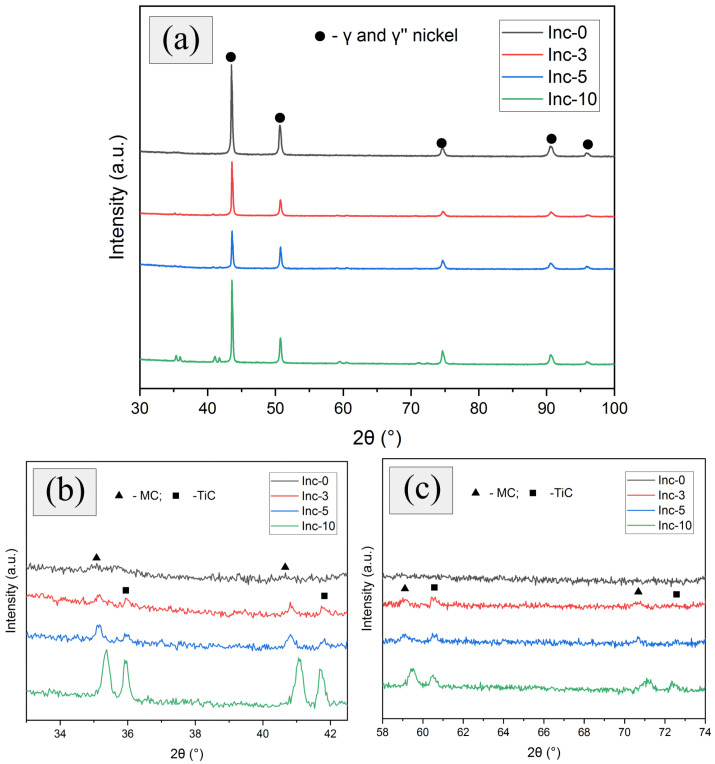
Diffractograms of samples after TO: (**a**) general view; (**b**,**c**) scaled areas of the graph.

The phase composition of the samples was determined using X-ray diffraction. Diffractograms of the samples after HT demonstrated the presence of peaks at angles characteristic of γ and γ″ phases of nickel (Figure 10a). In addition, in the scaled areas of the graphs (Figure 10b,c), peaks characteristic of the MC and TiC phases were observed, and the intensity of both types of these peaks increased with increasing TiC content in the material.

Titanium carbide could not be included in the MC phase designation because the peaks related to the TiC were observed only in the samples containing TiC (Inc-3, Inc-5, Inc-10), which cannot be said about the peak designated as MC, which was observed to some extent in each of the studied samples.

**Figure 11:**
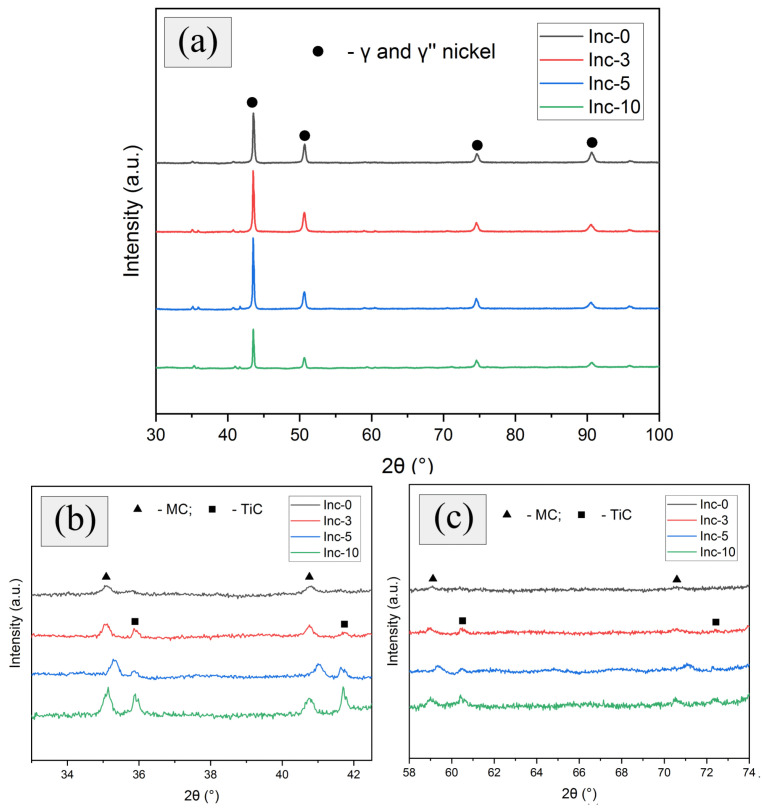
Diffractograms of samples after HIP and TO: (**a**) general view; (**b**,**c**) scaled areas of the graph.

Diffractograms of the samples after HIP and HT are presented in Figure 11.

In general, they could be described similarly to the diffractograms of the samples after HT, but it should be noted that the intensity of the MC-phase peaks in the Inc-0 sample increased as a result of HIP. Probably, the densification of the material due to the HIP contributed to the release of a relatively larger amount of this phase during the HT.

**Figure 12:**
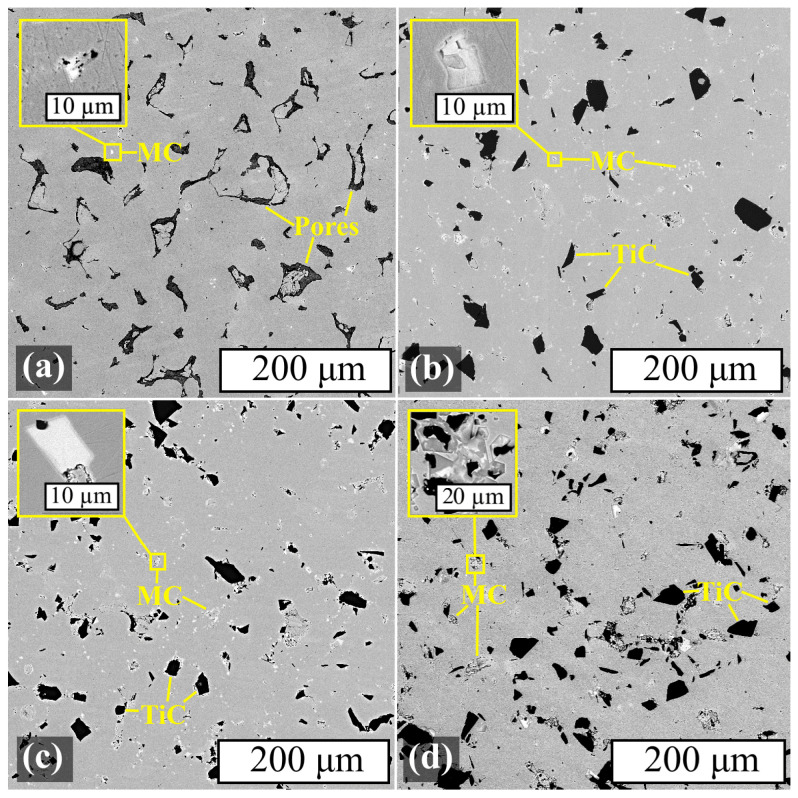
SEM images of samples after HT (BSE): (**a**) Inc-0; (**b**) Inc 3; (**c**) Inc-5; (**d**) Inc-10.

The microstructure study was carried out using SEM, and its results are presented in Figure 12.

The MC carbide phases that formed subsequent to heat treatment were predominantly located along the grain boundaries and in the proximity of TiC particles in the Inc-3, Inc-5, and Inc-10 samples. (Figure 12b–d). In the Inc-0 sample, the MC phase was released in much smaller amounts and was distributed chaotically (Figure 12a). It is also worth noting that the size of MC precipitates increased with increasing TiC content in the material, and in the Inc-10 sample these precipitates were in some cases comparable in size to the TiC particles.

**Figure 13:**
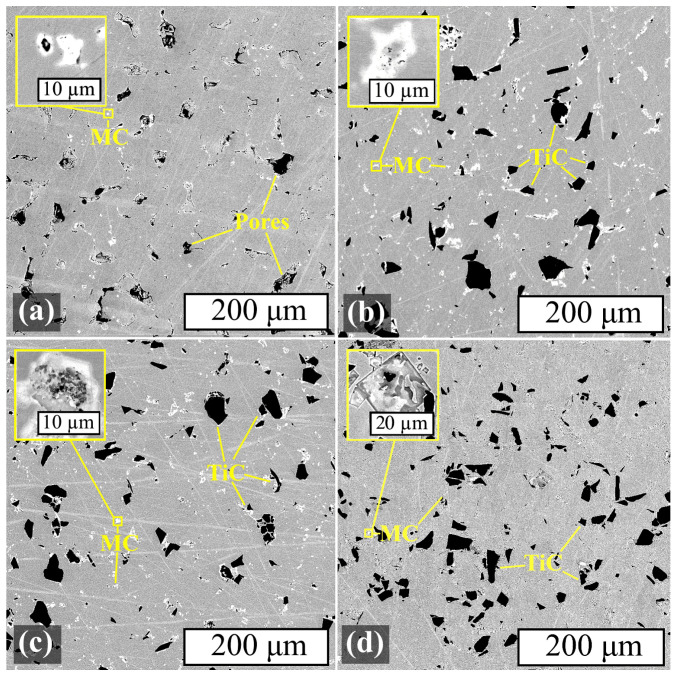
SEM images of samples after HIP and HT (BSE): (**a**) Inc-0; (**b**) Inc 3; (**c**) Inc-5; (**d**) Inc-10.

The microstructure of the samples after HIP and HT is presented in Figure 13.

The microstructure of samples Inc-3, Inc-5 and Inc-10 after HIP and HT could be described similarly to the samples subjected only to HT: the character of the MC phase distribution and the tendency to increase inclusions with increasing TiC content in the MMC remained the same. The Inc-0 sample was characterized by a slight decrease in porosity, as well as a noticeable increase in the amount of the MC phase, which is consistent with the XRD results.

**Figure 14:**
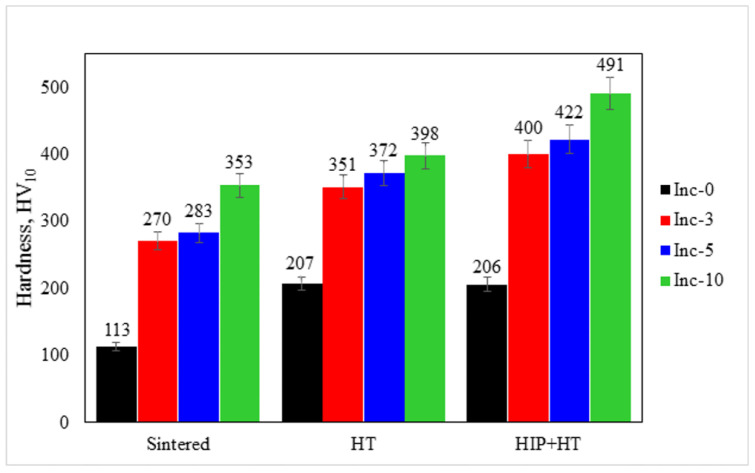
Results of hardness measurement of samples.

Hardness testing was performed on sintered samples and samples that had undergone two distinct post-treatment procedures: HT and HIP+HT. The measurement results are presented in Figure 14.

In the sintered state, the addition of TiC particles and the subsequent increase in its content led to an increase in hardness. The heat treatment contributed to the increase of hardness in all investigated samples due to the MC phase precipitating as a result of aging. The addition of the HIP step before HT had a favorable effect on the hardness of the samples containing TiC particles (Inc-3, Inc-5, and Inc-10). The Inc-0 sample, which did not contain added TiC, was characterized by a slight decrease in hardness when adding the HIP step to the post-processing mode. The low hardness values of the Inc-0 samples were probably due to their high porosity, including after HIP, which did not play a significant role in the densification of these samples.

Tensile tests were carried out at room temperature for two groups of specimens: after HT and after HIP+HT. The test results are presented in Table 4.

The test results showed that the addition of TiC to the matrix of Inconel 718 contributed to the increase of strength properties in all samples subjected to heat treatment. The best results after heat treatment were demonstrated by the Inc-3 samples, which were characterized not only by an increase in ultimate strength and yield strength, but also by an increase in relative elongation. Increasing the TiC content to 5 and 10 wt% significantly decreased the ductility, apparently due to too many carbide particles leading to embrittlement.

The addition of the HIP step prior to the HT resulted in an improvement in the mechanical properties of all samples containing added TiC. The best results after HIP and HT were still characterized by the Inc-3 samples, which had high strength and satisfactory ductility.

In contrast, the properties of the Inc-0 samples slightly deteriorated after the addition of the HIP step before the HT. It is worth noting that the tensile test results are in good agreement with the hardness measurements, which showed a deterioration in the hardness of the Inc-0 samples with the addition of the HIP step before the HT. The HIP did not promote significant densification in the Inc-0 samples, so even after its application, the samples were characterized by significant porosity. At the same time, HIP led to significant grain growth for this type of sample—from 34 to 40 μm (about 18%), which, together with the residual porosity, probably led to lower mechanical properties.

Given the results of room temperature tensile tests, which showed improved properties after the addition of the HIP step before HT for all TiC-containing samples, it was decided to perform the elevated temperature tests only for the group of samples with post-treatment including HIP and HT. The results of the tensile tests conducted at 700 °C are presented in Table 5.

Inc-3 samples showed the best mechanical properties, including high strength and satisfactory ductility at elevated temperatures. The yield strength and tensile strength values for the Inc-3 samples exceeded those of the corresponding characteristics presented in the results of a study [37], in which 1 wt.% TiC particles of a similar particle size distribution and morphology were added to a matrix of Inconel 718 superalloy. This suggests that a moderate increase in the carbide strengthening content in the metal matrix can effectively enhance the high-temperature properties of the composite material.

For all tested samples, an inverse relationship between ductility and TiC content in the composite material was observed. Inc-10 samples tested at elevated temperatures also exhibited high strength, but noticeably reduced ductility.

Inc-0 samples, similar to the results of the room temperature tests, had relatively low mechanical properties; their failure had a clearly pronounced brittle character, which is probably due to the high porosity of these samples.

**Figure 15:**
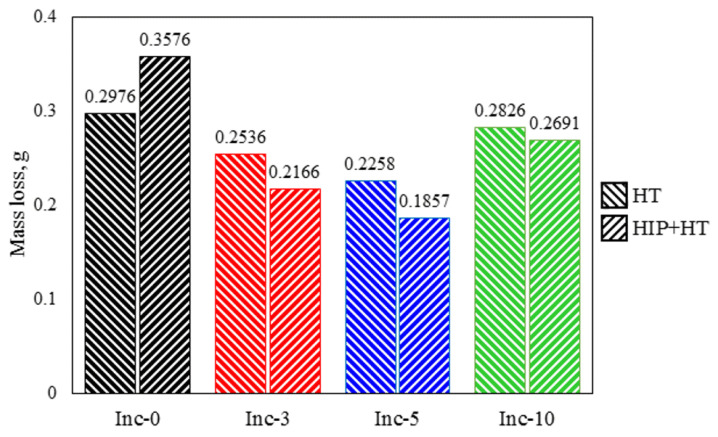
Total mass loss in abrasive wear tests.

The outcomes of the abrasive wear tests are illustrated in Figure 15.

The Inc-5 sample with post-treatment, including HIP and HT, was characterized by the lowest mass loss as a result of two abrasion cycles, i.e., it had the best wear resistance. These findings align with the outcomes of the tribological properties study of Ni/TiC composites presented in [48], which demonstrated that the incorporation of 5 wt.% TiC into the nickel matrix already resulted in a notable reduction in the coefficient of friction (approximately 23%).

Notably, increasing the TiC content of the composite material to 10 wt% decreased its wear resistance. This was presumably due to insufficient bond strength between the TiC particles and the matrix, leading to the carbide particles crumbling out during testing.

Friction is a synergetic result of bond strength, microstructure quality, and hardness [16], so each of these factors is extremely important. The Inc-10 sample had the highest hardness and was characterized by a defect-free structure; however, the bond strength between the TiC particles and the matrix in these samples was probably insufficient. The results indicate that it is possible to further optimize the properties of the Inc-10 composite by changing sintering modes, which represents a promising direction for future research. Investigation of alternative sintering conditions may enable performance improvements for this composite and expand its potential applications.

The Inc-0 sample had the worst wear resistance after HIP and HT. It is also worth noting that for the Inc-0 sample, there was an increase in abrasion weight loss after addition of the HIP step before HT, which agrees well with the results of tensile tests and hardness measurements that also showed a slight decrease in properties with post-treatment including HIP.

## 4. Conclusions

The titanium carbide (TiC) content within the Inconel 718/TiC composite material, produced via binder jetting additive manufacturing, exerted a considerable influence on both the processing parameters and the resulting mechanical and tribological properties of the composite.

1. An increase in the TiC content of the composite material resulted in the need for increased drying intensities during the binder jetting additive manufacturing process. This adjustment was necessary to offset the slow binder evaporation phenomenon, which resulted from the reduction in thermal conductivity of the powder mixture as the TiC content increased. This in turn required higher temperatures and extended drying periods.
2. The sintering modes were found to vary in accordance with the TiC content of the composites. The optimal sintering conditions, which maximized density while preserving the original geometry, were determined to be 1270 °C for 10 h for the Inc-0 and Inc-3 samples and 1280 °C for 5 h for the Inc-5 and Inc-10 samples.
3. The hardness values exhibited by the composite materials demonstrated a direct correlation with the reinforcement fraction.
4. The Inc-3 samples, after hot isostatic pressing (HIP) and heat treatment (HT), exhibited superior tensile properties, inclusive of performance at elevated temperatures, characterized by high strength and commendable ductility. Conversely, increasing the TiC content resulted in the embrittlement of the composite material.
5. The highest wear resistance was observed in the Inc-5 samples following HIP and HT. An increase in TiC content resulted in a reduction in tribological properties.

## Figures and Tables

**Table 1:** Designations for the investigated powders.

Powder	Designation
Inconel 718	Inc-0
Inconel 718 + 3 wt.% TiC	Inc-3
Inconel 718 + 5 wt.% TiC	Inc-5
Inconel 718 + 10 wt.% TiC	Inc-10

**Table 2:** Powder size distribution.

Powder	d_10_, μm	d_50_, μm	d_90_, μm
Inc-0	29.6 ± 1.0	49.7 ± 1.1	80.0 ± 1.3
Inc-3	21.6 ± 0.5	42.1 ± 0.9	73.1 ± 1.7
Inc-5	19.3 ± 0.4	39.5 ± 0.9	69.1 ± 1.8
Inc-10	15.7 ± 0.3	38.1 ± 0.4	68.5 ± 0.8
TiC	3.9 ± 0.2	17.1 ± 0.3	36.8 ± 0.2

**Table 3:** Binder jetting process parameters.

Powder	Layer Thickness, mm	Binder Saturation, %	Bed Temp, °C	Dry Time, s
Inc-0	0.1	60	30	12
Inc-3	0.1	60	31	18
Inc-5	0.1	60	32	20
Inc-10	0.1	60	33	21

**Table 4:** Results of tensile tests at room temperature.

Material	YS, MPa	UTS, MPa	δ, %
Inc-0 HT	676 ± 7	821 ± 8	4.9 ± 0.1
Inc-3 HT	804 ± 8	971 ± 10	5.8 ± 0.2
Inc-5 HT	732 ± 7	849 ± 8	2.3 ± 0.1
Inc-10 HT	773 ± 7	852 ± 9	0.8 ± 0.1
Inc-0 HIP+HT	627 ± 6	752 ± 7	2.6 ± 0.1
Inc-3 HIP+HT	968 ± 10	1127 ± 11	9.4 ± 0.3
Inc-5 HIP+HT	927 ± 9	1040 ± 10	6.9± 0.2
Inc-10 HIP+HT	937 ± 9	996 ± 10	0.9 ± 0.1

**Table 5:** Results of tensile tests at 700 °C.

Material	YS, MPa	UTS, MPa	δ, %
Inc-0 HIP+HT	-	375 ± 4	0.0
Inc-3 HIP+HT	800 ± 8	877 ± 9	3.7 ± 0.2
Inc-5 HIP+HT	782 ± 8	823 ± 8	2.6 ± 0.2
Inc-10 HIP+HT	872 ± 9	876 ± 9	0.7 ± 0.1

## Data Availability

The original contributions presented in this study are included in the article, further inquiries can be directed to the corresponding author.
